# Toddler home math environment: Triangulating multi-method assessments in a U.S. Sample

**DOI:** 10.3389/fpsyg.2023.1105569

**Published:** 2023-02-21

**Authors:** Portia Miller, Leanne E. Elliott, Tamara Podvysotska, Chelsea Ptak, Shirley Duong, Danielle Fox, Linsah Coulanges, Melissa Libertus, Heather J. Bachman, Elizabeth Votruba-Drzal

**Affiliations:** ^1^Learning Research and Development Center, University of Pittsburgh, Pittsburgh, PA, United States; ^2^American Institutes for Research, Arlington, VA, United States; ^3^Department of Psychology, Kenneth P. Dietrich School of Arts & Sciences, University of Pittsburgh, Pittsburgh, PA, United States; ^4^Department of Psychology in Education, School of Education, University of Pittsburgh, Pittsburgh, PA, United States

**Keywords:** math (STEM), toddler age, home learning environment, measurement, methodology, home math environment

## Abstract

**Introduction:**

Current research has documented the home math environment (HME) of preschoolers and kindergarteners. Very few studies, however, have explored the number and spatial activities in which parents engage with children during their toddler years.

**Methods:**

This study examined the HME of 157 toddlers using several methodologies, including surveys, time diaries, and observations of math talk. Further, it examined correlations within and across data sources to identify areas of convergence and triangulation, and correlated HME measures with measures of toddlers’ number and spatial skills.

**Results:**

Findings showed that, in general, uses of different types of math activities, including both number and spatial, were intercorrelated within method. Across methods, there was high intercorrelation between the frequency of math activities reported on parent surveys and the diversity of types of math activities endorsed in time diary interviews. Parent math talk gleaned from semi-structured interviews functioned as a separate aspect of the HME; different types of math talk shared few intercorrelations with engagement in math activities as reported in either surveys or time diaries. Finally, several HME measures positively correlated with toddlers’ math skills.

**Discussion:**

Given extant research demonstrating that both math activities and math talk predict children’s math skills, our results stress the need for multimethod studies that differentiate among these HME opportunities.

## Introduction

Early mathematics skills lay the foundation for later math achievement as well as academic skills more generally (Duncan et al., 2007; Jordan et al., 2007, 2009). Many of these math skills start to emerge during the toddler years when children begin to develop the symbolic number and spatial skills that we often think of in traditional conceptions of math. In terms of numeracy, by age two, children begin to understand the meaning of number words. Initially, children understand that number words form a category of words separate from other categories such as color words, and they may be able to recite the count list without fully understanding the meaning of these words. Around 2.5 years of age, most English-speaking children understand the word “one” and correctly give one object when asked for one in contrast to two or three objects. Over the next months and years, children incrementally develop an understanding for subsequent number words (Wynn, 1990, 1992). Likewise, toddlers show a rudimentary understanding of spatial perspective taking and mental rotation, such as understanding that turning a shape toy may help it fit in the designated hole, though these skills continue to undergo refinement throughout childhood (see Newcombe et al., 2013). Additionally, knowledge of spatial language is displayed in infants before their first birthday, but expressive spatial vocabulary is usually not demonstrated until the third year of life (Pruden et al., 2004).

Children’s earliest environments can shape the development of their math skills, including their early interactions in the home with parents or other family members. A growing body of research addressing these opportunities for learning math, collectively referred to as the home math environment (HME), demonstrates that preschoolers’ and kindergarteners’ exposure to number and spatial concepts at home positively predicts math skills (Elliott and Bachman, 2018; Daucourt et al., 2021; Hornburg et al., 2021). Notably, however, few studies have examined toddlers’ exposure to math concepts at home. Understanding children’s HME in toddlerhood is particularly important given that, on average, toddlers spend more time in the home than do preschool- and school-aged children who spend a larger portion of their day in school settings. In this study, we examine different measures of the HME among toddlers, including surveys of math activities, time diary interviews, and observations of math talk, and assess how these measures relate. We compare measures of parent–child math activities, typically based on the frequency of specific activities or the diversity of different activities that children engaged in, and measures of how much parents talk about math during different semi-structured interactions with their children.

### Measures of the home math environment

Although a long history of research has examined the home environments of infants and toddlers, much of this work addresses how parents provide opportunities for cognitive stimulation more broadly (e.g., Bradley and Caldwell, 1984; Foster et al., 2005; Chazan-Cohen et al., 2009; Rodriguez and Tamis-LeMonda, 2011) or engage in specific activities to support reading and language skills (e.g., Schmitt et al., 2011; Kim et al., 2015; Linberg et al., 2020). In contrast, less is known about the types of activities at home that might support toddlers’ math learning. In this study, we use three methods of assessment of math activities with toddlers to help address this gap in the literature: traditional surveys, semi-structured observational tasks, and time diary interviews.

#### Parent surveys

Recent work with preschool- and kindergarten-aged children demonstrates that parents’ reports of the frequency with which they engage in math-related activities at home with their children in surveys is positively related to children’s math learning (see Daucourt et al., 2021, for meta-analysis). These positive links are primarily observed for activities that include number content, such as playing board games or counting objects (LeFevre et al., 2009, 2010; Siegler and Ramani, 2009). Math activities also include activities that have a spatial reasoning component, like playing with puzzles, building with blocks, or measuring objects, though these activities tend to be reported less frequently among parents of preschoolers than number activities (Zippert and Rittle-Johnson, 2020), and their links to children’s early math skills are much less consistent than links between number activities and math (Hart et al., 2016; Purpura et al., 2020).

#### Time diaries

In contrast to traditional survey measures, time diaries offer a novel method of collecting data on families’ day-to-day activities, where adults provide minute-by-minute reports of their activities over the course of a day (Phipps and Vernon, 2009). In past research using this approach, researchers have captured the amount of time children spend in various cognitively stimulating activities, such as reading or structured playtime (e.g., Hofferth and Sandberg, 2001; Fiorini and Keane, 2014). However, math activities may occur more sporadically throughout the day, and so additional probing for these interactions during interviews may be needed to obtain a more accurate view of number and spatial activities occurring at home. A recent study with parents of preschoolers found that very few parents spontaneously reported engaging in math activities during the day, but when asked whether specific activities occurred, almost all parents had engaged in some math activities with their children (Bachman et al., 2020). In other words, many math activities may occur in the context of other interactions, and parents tend to only report the larger activity within which the math activity took place. For example, parents may report baking with their children and not mention that they counted and compared measuring cups, but when asked about these specific behaviors, they report having engaged in these math activities. This additional probing may be particularly important for accurately measuring the frequency of math activities and will give rise to higher incidence of math activities than minute-by-minute reports of activities would suggest.

Although both survey measures and these probes embedded in time diaries rely on parental report of similar activities at home, time diaries may have some methodological advantages, including stronger ecological validity and fewer issues of recall bias. As an additional advantage, time diaries can assess duration of math activities, i.e., time in minutes spent engaged in activities, in a way that questionnaires do not because these typically focus on the number of days per week. On the other hand, by only asking about a select few days, the scale of the time diary reports is also much narrower than survey measures that often ask parents to report on larger periods of time, such as the prior week or two or even a whole month. Our past work with preschoolers suggests high levels of concordance between survey and time diary reports of math activities at home (Bachman et al., 2020), a finding we seek to extend here to a younger sample of children.

#### Parent math talk

As an alternative to parent reports of math activities, many researchers have measured math talk by examining how much and in what ways parents and children discuss number and spatial content, either during structured observational tasks that are math-related (e.g., Ramani et al., 2015; Leyva et al., 2017) or during naturalistic play or other everyday activities (e.g., Levine et al., 2010; Elliott et al., 2017). Much of the past math talk literature focuses on the frequency of children’s exposure to number talk, or parents’ use of number words, during the preschool and kindergarten years and how this number talk predicts children’s number knowledge and math skills more generally (e.g., Mix and Cheng, 2012; Ramani et al., 2015; Elliott et al., 2017), with some nuances in the types of number talk and ways number talk is used (e.g., pairing the count list with cardinal values, or using larger number words). Similar patterns of associations are seen for children between one and 3 years of age, such that exposure to number talk in the toddler years predicts preschoolers’ understanding of cardinality (Levine et al., 2010). Parents’ use of number talk is likely context-dependent, as one study showed that number talk in a lab setting and observed at home were not significantly related (Thippana et al., 2020), Similarly, parents number talk tends to vary across different structured activities (Ramani et al., 2015; Zippert and Rittle-Johnson, 2020). Thus, in the present study we examine two contexts that may elicit number talk: a book reading task and a pretend grocery store activity.

Compared to number talk, less research has examined parents’ discussions of spatial content with their young children, but the extant evidence demonstrates that the frequency of parents’ use of spatial terms is positively related to children’s spatial skills, possibly through children’s own spatial vocabulary (Pruden et al., 2011; Polinsky et al., 2017; Casasola et al., 2020). Moreover, we recently showed that the complexity of parents’ spatial talk as measured by the mean length of spatial talk utterances during a spatial activity predicted preschoolers’ growth in spatial skills (Fox, n.d.). Much like number talk, parents’ use of spatial talk varies depending on context and activity but in general is more frequent among activities that are inherently spatial, such as when building with blocks (Ferrara et al., 2011; Verdine et al., 2019; Zippert and Rittle-Johnson, 2020; Fox, n.d.). Although much of this work examines spatial talk frequency during the preschool years (age 4–5), more frequent parent spatial language use when children are between one and 3 years of age also predicts children’s later spatial skills (Pruden et al., 2011). Here, we examine parents’ use of spatial talk during a puzzle activity with their toddler.

### Associations between the home math environment and children’s math skills

Importantly, past work with children in early childhood demonstrates developmental differences and inconsistencies in the frequencies of home math activities as well as math talk and their relations to children’s math skills, which could be due in part to the different methods used to measure HME (e.g., Hart et al., 2016; Thompson et al., 2017). For instance, Thompson et al. (2017) examined associations between HME, measured using survey methods, and math skills for 3- and 4-year-olds. In that study, correlations between HME and math were significant among the 4-year-olds but non-significant for 3-year-olds. However, a meta-analysis synthesizing results of more than 68 studies found that links between HME did not vary across age, though the youngest children sampled were 3-years-old (Daucourt et al., 2021). With respect to math talk, a study by Levine et al. (2010) showed that parental number talk at home to 2- to 3-year-old children predicted children’s cardinality skills when they were four, while other studies do not find longitudinal associations between parents’ frequency of math talk and children’s math skills (Son and Hur, 2020; Fox, n.d.).

The discrepancies in previous studies exploring the link between HME and math skills in early childhood highlight the importance of additional research capitalizing on multiple methods to characterize the HME during toddlerhood. Indeed, the HME may be especially important in the development of math skills for toddlers, compared to preschoolers and older children, because once children enter preschool and elementary school, schooling effects contribute to math skills as well. Yet, few studies have examined the number and spatial activities in which parents engage with children during the toddler years. Thus, the present study provides rich description of toddlers’ home math environments derived from multiple, interdisciplinary methods, including parent-reported questionnaires, semi-structured observational tasks, and time diaries. Additionally, this study will examine whether there is convergence within and across multiple modalities of HME measurement and provide exploratory correlations among HME measures and toddlers’ early number and spatial skills.

### The current study

Although talk about math concepts is likely to occur more frequently during activities that are explicitly math-related, conversations about number and spatial concepts can occur in everyday interactions and activities as well (Anderson et al., 2004; Susperreguy and Davis-Kean, 2016; Pruden and Levine, 2017; Thippana et al., 2020). As such, frequencies of math talk and math activities likely reflect distinct components of the overall HME (see Hornburg et al., 2021). Past work examining math talk and math activities in particular yields a mixed pattern of findings, with some studies demonstrating significant associations across measures (e.g., Thippana et al., 2020) where others find no correlations (e.g., Mutaf Yildiz et al., 2018). On the one hand, math activities are more likely to elicit math talk suggesting that more frequent math activities should also be associated with more math talk. However, in most of the published work, math talk is measured during non-math activities (e.g., free play, mealtimes), which may evince different amounts of math talk. Previous research has reported that parents’ number talk during non-math activities is associated with parents’ education and children’s gender, while parents’ number talk during math activities is unrelated to these factors (Thippana et al., 2020). Thus, it is possible that different factors influence when and how parents engage in math talk with their children in different activities resulting in different associations with frequencies of parent-reported math activities. In our own work with parents of preschoolers, we find little evidence of associations between the frequencies of parents’ spatial and number talk and their reported spatial and number activities, either through survey measures or time diaries (Bachman et al., 2020). In this study, we aim to extend these analyses to a younger sample of children and consider how parents of toddlers engage in activities and have conversations related to math concepts with their children. Furthermore, we look at how these different measures of HME correlate with toddlers’ early number and spatial skills.

## Methods

### Participants

This study draws data from the Parents Promoting Early Learning (PPEL) study, a longitudinal study of 157 parents and their toddlers (74 boys) studying parent factors and home experiences that bolster early math learning in toddlerhood. Children in this study were on average 2 years and 7.86 months old (SD = 2.47 months), ranging from 2 years and 4 months to 3 years 3 months of age. Participating parents were predominantly mothers (*n* = 149), but fathers (*n* = 8) also participated in this study. Most parents identified as non-Hispanic White (76%), with others identifying as Black (12%), Hispanic/Latino (3%), Asian (2%), or another race (3%). Parents also tended to be highly educated (76% had at least Bachelor’s degree) and married (80%). Based on household income and family size, 22% of families were classified as low-income (i.e., earnings below 200% of the poverty line), 32% as middle-income (i.e., earning between 200 and 399% of the poverty line), and 46% as high-income (i.e., earnings 400% and above of the poverty line). Descriptive statistics are shown in Table 1.

**Table 1:** Descriptive statistics of the sample demographics.

	M(SD)/%
Child age (in Years)	2 yrs. 7.86 mths (2.5 mths)
Child sex (Male)	47%
Parental family status (Married)	80.3%
Parents’ race	
White non-Hispanic	76%
Black	12%
Asian	2%
Hispanic/Latino	3%
Other/multiracial	3%
Prefer not to answer	3%
Parents’ education (Bachelor and higher)	76%
Parents’ income	
Low income	22%
Middle income	32%
Upper income	46%

### Procedure

Due to the COVID-19 pandemic, this study was conducted entirely online through a combination of video conferencing calls, phone calls, and online surveys. Families were recruited from the greater Pittsburgh, Pennsylvania metropolitan area through online postings and advertisements on social media (e.g., Facebook), online research participant registries, and flyers distributed through local community organizations, preschools, and in parks. Study materials were delivered to families’ homes, including assessment materials, toys, paper surveys, and, if needed, a laptop and Wi-Fi hotspot. Families participated in two Zoom calls with research assistants for approximately 30 min per session. During Zoom calls, children completed cognitive assessments, and the parent and child engaged in several play-based semi-structured interactions. The order of testing sessions was fixed, but the order of tasks within testing sessions was counterbalanced. All Zoom calls were recorded for later scoring of cognitive assessments and coding of parent–child interactions. Sessions were conducted, on average, about 1 week apart, though times between Zoom sessions ranged from as little as 1 day to as much as almost 3 months depending of families’ schedules.

Parents also received two phone calls on separate days to complete time diaries reporting on the previous days. Calls were scheduled so that parents reported about activities on a work day and a non-work day. Finally, parents were sent an online survey including questions about demographic information and home learning activities. All research activities were approved by the local Institutional Review Board, and all parents gave written informed consent to participate in the study prior to completing any research activities. Families were compensated up to $100 for participating in the study. Data used in this study were collected from children and parents during the Zoom calls, phone calls, and electronic questionnaires. Measures of math activities were drawn from the online survey and time diary interviews. Measures of math talk were drawn from the semi-structured observations.

### Measures

#### Home math activities

Parents completed questionnaires designed to assess the frequency of number and spatial activities at home over the last month (LeFevre et al., 2009). Parents were given a list of math activities in the home and asked to report how frequently they engaged with their children in each on a scale from 1 (“did not occur”) to 5 (“almost daily”; LeFevre et al., 2009). These items were drawn from the work of LeFevre et al. (2009, 2010) and some were adapted to make them applicable to toddlers, include activities like “counting objects,” “playing board games with die or a spinner,” “learning simple addition,” and “measuring ingredients when cooking.” In our prior work, we identified three factors of numeracy activities, including those that address basic numeracy concepts (e.g., categorizing objects, identifying the meaning of number words), applications of number concepts (e.g., measuring ingredients while cooking, talking about money while shopping), and written numerals (e.g., reading number storybooks, playing with number toys; Elliott et al., 2023). Parents’ responses were averaged to form these three number composites: number concepts (4 items, α = 0.69); written numerals (4 items, α = 0.78); and number applications (6 items, α = 0.66). Similarly, responses on 5 items categorized as spatial activities were averaged into two separate composite scores tapping shape activities (3 items, α = 0.61) and building activities (2 items, α = 0.63). Higher scores indicate more frequent engagement with the number and spatial activities.

#### Math talk

Parents and children were observed while engaging in three semi-structured tasks designed to elicit either number or spatial talk. To elicit number talk, researchers provided dyads with developmentally appropriate toys for pretend grocery shopping, including a shopping basket, cash register, pretend money, and a play set of food items. Parents were instructed to play with these toys with their child as they normally would for 8 mins. Previous research has shown that a pretend grocery store can elicit high levels of math-related talk (Elliott et al., 2017). Parents and children also completed a shared book reading task. Dyads were given a wordless picture book created by the study team and designed to elicit number talk (Ginsburg et al., 2018). Parents were asked to read the book with their child and were prompted to finish the book reading after 3 mins. To elicit spatial talk, parents and children completed a magnet board puzzle task during which they were given magnets of various colors and shapes and asked to create an animal. Studies show that “guided play” tasks like this elicit high frequencies of spatial talk in parents and children (Ferrara et al., 2011). Dyads took up to 8 mins to complete the puzzle activity.

Each task was videotaped, transcribed verbatim at the utterance-level, and checked by trained research assistants. An utterance was defined as any language input from an individual speaker (either parent or child) that is bounded by silence of at least 2 s, a speaker transition, or a grammatical closure, e.g., a terminal punctuation mark such as a period (Pan et al., 2004). Transcriptions from direct observation tasks were coded for the quantity of parents’ number and spatial talk. Specifically, the *total number of number utterances* during the grocery and book tasks was calculated, and then each number utterance was coded for the utterance content. We identified several types of number talk content that occurred during the grocery and book tasks, three of which were included in these analyses given their relatively high frequencies of use: (1) identifying *number symbols*; (2) *counting*; and (3) *labeling set sizes*. Number utterances involving comparing magnitude, ordinal relations, arithmetic, and patterns were coded but not used in this study because they were observed at such low frequencies (means ranging from 0.03 to 0.31 and medians of zero). The *total number of spatial utterances* during the puzzle activity was also calculated, and each spatial utterance was also coded for the utterance content. We examined three types of spatial talk that frequently observed during the puzzle activity: (1) discussing *shapes*; (2) *locations, directions, and orientations*; and (3) *deictics* (words whose meanings depend on the speaker’s point of view, i.e., “here,” “there,” “where”). Two additional types of spatial talk were observed, but in such low rates that we were unable to include them in analyses. These were spatial dimensions and spatial properties, with the mean number of utterances of these types during the puzzle activity equaling 0.4 and a median of zero.

Coders for both number and spatial talk included graduate students, postdoctoral researchers, undergraduate research assistants, and full-time research staff. Following standard practices (Hallgren, 2012; Chorney et al., 2015), inter-rater reliability on the number and spatial codes for each task was assessed for over 20% of the sample by calculating the kappa statistics for each code between pairs of coders in identifying and categorizing each math talk utterance. Reliability was calculated at the utterance level from the full set of utterances. For example, when calculating reliability for utterances involving counting, cases of disagreement could include times where one coder did not identify the utterance as number talk at all and the second coded it as counting as well as times where one coder identified the utterance as a different type of number talk than counting when the second coded it as counting. This was the most conservative approach, since coders would have to both correctly identify an utterance as number talk and code it in the correct category of content or utterance type in order to count as agreement. The initial coder’s classification was used in the case of disagreements. For number talk, coders examined a total of 2,014 utterances that were flagged as potentially number-related (based on their inclusion of number words or elicitations). There was a moderate to strong degree of reliability in labeling utterances across number talk categories (κ = 0.83–0.91; McHugh, 2012). For spatial talk, coders examined a total of 6,083 utterances. The coding of our spatial content codes also showed strong to almost perfect levels of agreement (κ = 0.86–0.93).

#### Time diary reports of diversity and duration of math activities

The diversity and duration of math activities was measured using the time diary interviews. Parents completed two time diary interviews over the phone collected using a modified format of the American Time Use Survey (ATUS; United States Bureau of Labor Statistics, 2016) during which they reported all activities carried out by parents and children over a work day and a non-work day. If the parent worked every day or was not employed, the time diaries were completed to reflect activities on a weekday and a weekend day. The phone interview occurred 1 day after the target day to facilitate accurate recollection of activities.

After parents reported the activities, they were surveyed at the end of the phone interview about the formal and informal home learning practices that occurred the prior day. These questions modeled survey items in LeFevre et al. (2009) work. These questions asked for occurrence of different activities, and if the activity occurred, the duration of the activity (i.e., parent reported time child spent engaged in an activity). Specifically, parents were asked whether a math activity occurred the previous day and were provided with a list of examples of this activity. If the parent said the larger category activity occurred, they were asked about the occurrence of a series of subcategory activities, giving a yes/no response, and to provide an approximate amount of time the child spent engaging in the activities. For example, parents were asked, “Did your child spend any time working or playing with numbers (both written and spoken)? This would include identifying names of written numbers (e.g., in magazines or in an elevator), identifying meaning of numbers (e.g., “how many is three”), or playing with toys that involve numbers (e.g., number fridge magnets, number stamping activities, foam numbers, etc.)?” If parents responded “yes” to working or playing with numbers, they were asked about occurrence and duration of all activities included in the broader category. The full list of items contained in the interview are listed in Table 2. From this list of items, we created measures of the *diversity of number activities*, which summed all number activities in which parents reported children engaged, and the *diversity of spatial activities*, which summed all spatial activities in which parents reported children engaged. We also summed across these measures to create a measure of the *total diversity of math activities*. Finally, we created a *duration of math activities* measure representing the total minutes in which children were engaged in all math activities.

**Table 2:** Academic stimulation phone interview items.

	No	Yes	How long it lasted
MATH			
**Did your child spend any time working or playing with numbers (both written and spoken)? This would include…**			
Identifying names of written numbers (in magazines, in the elevator)			
Identifying meaning of numbers (“How many is three?”)			
Playing with toys that involve numbers (e.g., number fridge magnets, number stamping activities, foam numbers, etc.)			
**Did your child spend time counting?**			
Counting objects (e.g., counting child’s fingers, counting number or jumps or steps while playing, counting beads)			
Reciting numbers (e.g., 1,2, 3, 4,…)			
Counting down (10, 9, 8, 7, …)			
**Did your child categorize or compare objects? So things like…**			
Categorizing or organizing things by a common feature such as size, color, or shape (e.g., sorting blocks by color)			
Making collections (e.g., rocks, toy animals)			
Comparing things (e.g., by size, weight)			
**Did your child talk with others about shapes or play with shapes?**			
Playing with a shape sorter			
Talk about shapes or identify shapes? (e.g., What shape is this? Where do you see a square?)			
**What about using math while shopping or cooking? So things like…**			
Talking about money when shopping or while playing grocery shopping (e.g., “which costs more?”)			
Measure ingredients while cooking or while pretending to cook (e.g., “We need two eggs and one stick of butter,” “Can I have one more chocolate?”)			
Compare food while eating (e.g., “who has the bigger plate, you or Mommy?” “Which of your strawberries is bigger?”)			
**Did someone talk to your child about dates or times? So maybe….**			
Have conversations about time concepts (morning, afternoon, night, today, tomorrow, yesterday, “two days until your grandma comes”)			
Timing (e.g., timing how long it took the child to complete a task, timing how many minutes)			
**What about books or activities that involve math? This could include…**			
Using rhymes that involve numbers (“1, 2, buckle my shoe” “Six little ducks went out one day…”)			
Reading number storybooks			
Reading books to teach shapes			
Reading books to teach numbers (Counting picture books)			
**Did your child play games that could involve math? This would include…**			
Playing board games or cards that involve shape matching or counting			
Playing with puzzles			
Building Lego, blocks or construction set (Duplo, Megablocks etc.)			
**Did your child use any video, computer games, or electronic toy focused on numbers or math concepts yesterday? Did you…**			
Use educational software			
Play other videogames			
READING			
**Did your child spend time reading with someone yesterday? This would include…**			
Reading a story together.			
Reading signs or other non-book items with words on them.			
Child looked at books independently.			
**Did your child engage in story telling with someone? This can include…**			
Outside of book reading, telling a story to your child			
Your child telling you a story that involved a sequence of events (e.g., beginning, middle, and end)?			
**Did your child play sound or word games? This includes**			
Play games with beginning sounds of words (e.g., cat starts with “cuh,” Which word starts with /s/ like “snake”?)			
Play rhyming games with your child?			
Recite nursery rhymes that do not involve numbers?			
Sing songs with your child?			
**Did your child engage in activities that involve letters? This includes**			
Practice naming the letters of the alphabet.			
Ask your child to identify letters.			
Play with alphabet toys at home.			
Identify the sound of letters of the alphabet (e.g., asking “what sound does the letter D make?)			
Point out letters or words (e.g., directing your child’s attention to words on street signs)			
**Did your child use any video, computer games, or electronic toys focused on letters, letter sounds, or reading? Did you…**			
Use educational software			
Play other videogames			

#### Children’s math skills

Children’s *counting ability* was assessed using a task that asked children to count out loud on their own. If a child did not start counting independently after being asked by the researcher, the researcher would count up to two to help (i.e., “One, two, …. what comes next?”). Children were allowed to correct themselves or start over again if they indicated that they made an error. They were stopped once they made a mistake or reached 100. Children’s scores on this task were recorded as the highest number to which they were correctly able to count.

*Spatial reasoning* was assessed using the Point-to-Spatial-Relations task (Casasola, 2005), which measures children’s spatial relation language comprehension. For each of seven trials, toddlers were shown PowerPoint slides (*via* Zoom screen share) of a stuffed animal posed with a red plastic cup. Children were prompted to identify the picture that matched the spatial relation between the stuffed animal and cup described by the researcher. The following spatial language terms were included: “on top of,” “under,” “between,” “in front of,” “behind,” “in,” and “next to.” A proportion score was created for each toddler by summing the total number of correct responses and then dividing by the total number of trials completed by the child.

### Analysis plan

To address our research aims, we examined patterns of correlations within each data source (i.e., parent questionnaires, math talk, and time diaries) and then across three data sources to identify areas of convergence and triangulation. Finally, we correlated children’s early counting and spatial reasoning skills with the HME measures. Prior to running correlations, we addressed missing data in our sample. Level of missingness varied depending on the data source, ranging from no missing data for time diaries observations to a high of 13.4% missing (21 missing observations) for parent questionnaire data. In addition, some of the time diary duration entries were highly skewed and appeared to be errors in reporting (e.g., a report of almost 1,000 min or more than 16 h of math activities over 2 days). To address this, we recoded as missing any time diary duration measure that was greater than three standard deviations above the sample mean. Missing data were imputed using the multivariate imputation by chained equations (MICE) package in R to create 40 imputed datasets (Van Buuren and Groothuis-Oudshoorn, 2011). Our final analytic sample totaled 157 observations across all correlations.

## Results

### Parent surveys of home math activities frequencies

Table 3 presents descriptive statistics on parent responses to the survey items assessing frequency of math activities. According to the survey, children engaged in all types of math activities examined fairly frequently, with the lowest endorsed category being activities involving *number applications* (mean of 2.4 on a scale of 1 to 5). The other four categories, *number concepts*, *written numerals*, *shape activities*, and *building activities*, were reported more frequently, with means ranging from 3.2–3.8. Table 4 presents the correlations between the frequencies of math activities reported on the parent questionnaire. Engagement in all types of math activities captured in the parent questionnaire were significantly correlated, with correlations ranging from 0.17 to 0.62. Looking specifically at correlations within subdomains, number activities were moderately to strongly correlated with one another, with the strongest correlation observed between *number concepts* and *written numerals*. The two spatial activities composites, *shape activities* and *building activities*, also correlated modestly with each other. Significant correlations existed across number and spatial domains of activities. Indeed, the strongest correlation between math activities was observed between activities in different domains; engagement in *written numerals* and *shape activities* were the most highly correlated of all math activities reported.

**Table 3:** Descriptive statistics for parental math support measures based on unimputed data.

	n	M	SD	Min	Max
Home math activities scale					
Number concepts (q)	150	3.76	0.87	1	5
Written numerals (q)	150	3.38	0.99	1	5
Number applications (q)	152	2.41	0.83	1	5
Shape activities (q)	152	3.23	0.91	1	5
Building activities (q)	152	3.72	0.99	1	5
Number talk					
Total number utterances (o)	157	25.39	17.75	0	81
Number symbols (o)	157	3.18	5.11	0	35
Counting (o)	157	6.14	7.23	0	50
Labeling sets (o)	157	16.07	11.11	0	53
Spatial talk					
Total spatial utterances (o)	152	31.96	14.58	5	73
Shapes (o)	152	10.48	7.97	0	36
Locations, directions and orientations (o)	152	12.88	8.87	0	52
Deictics (o)	152	13.98	8.16	1	42
Time Diary (TD) codes diversity of total math activities (td)	157	13.94	8.53	0	44
Diversity of spatial activities (td)	157	3.43	2.30	0	11
Diversity of number activities (td)	157	10.97	5.83	0	26
Minutes of math activities (td)	151	129.76	106.02	0	580

(q), survey of home math activities, (o), math talk content from the semi-structured observations, (td), math activities reported by parents in the time diary interview.

**Table 4:** Pair-wise correlations among number and spatial activities at home as reported on parent questionnaire.

	1	2	3	4
1. Freq. number concepts				
2. Freq. written numerals	0.53***			
3. Freq. number applications	0.38***	0.41***		
4. Freq. shape activities	0.47***	0.62***	0.33***	
5. Freq. building activities	0.34***	0.27***	0.17*	0.34***

*p < 0.05, **p < 0.01,***p < 0.005.

### Observations of math talk during semi-structured interactions

#### Number talk

As can be seen in Table 3, during the grocery and book tasks, the most frequent number talk involved *labeling set sizes*. On average, parents labeled set sizes about 16 times. Relatively less math talk involved *counting* and identifying *number symbols*. The intercorrelations between number talk utterances across tasks were positive and significant. As is shown in Table 5, talk concerning *labeling set sizes* was moderately correlated with *number symbols* and *counting* talk. Also, the total amount of number utterances was correlated with each of the three number talk content areas, with moderate correlations between total number utterances and *number symbols* talk and *counting* and very high correlations between total number talk and *labeling sets*.

**Table 5:** Pair-wise correlations among number and spatial talk taken from semi-structured observations.

	1	2	3	4	5	6	7
1. Talk number symbols							
2. Talk counting	0.20*						
3. Talk labeling sets	0.38***	0.35***					
4. Total number utterances	0.61***	0.68***	0.88***				
5. Talk shapes	0.01	0.06	0.09	0.08			
6. Talk locations, directions, orientations	0.13	0.11	0.30***	0.27***	0.34***		
7. Talk deictics	0.19*	0.10	0.16	0.19*	0.06	0.41***	
8. Total spatial utterances	0.19*	0.1	0.32***	0.32***	0.56***	0.83***	0.68***

*p < 0.05, **p < 0.01,***p < 0.005.

#### Spatial talk

As is shown in Table 3, the amount of spatial talk across content areas was highly similar, averaging about 10–13 utterances per type. Types of spatial utterances were positively and significantly correlated, except for *shapes* and *deictics* utterances (Table 5). Moderate correlations were observed between *locations, directions and orientation* with *shapes* and *deictics*. Total spatial utterances were correlated with the specific content area utterances, with correlations ranging from 0.56 to 0.83.

#### Intercorrelations among number and spatial talk

In addition to within-number and within-spatial domains associations, we also analyzed whether parents who used more number talk also used more spatial talk during the observational tasks (Table 5). In terms of overall number and spatial talk, there was a positive correlation between *total number utterances* and *total spatial utterances*. Looking at specific content areas across domains, this correlation was driven by the correlation between *labeling sets* and talk involving *locations, directions, and orientation*. Parents who labeled more set sizes in the grocery and/or book tasks also tended to talk more about locations, directions, and orientation in the puzzle activity. There was also a small but significant positive association between *number symbols* utterances and *deictics* utterances. No other cross-domain correlations were observed when looking at the specific number and spatial talk content areas.

### Diversity and duration of math activities based on parent time diary interviews

As noted in the methods, we used three measures from the time diary interviews that captured the diversity of number and spatial activities and the duration of math activities in which children engaged across the 2 days captured by the time diary. Descriptive statistics on time diary variables are shown in Table 3. On average, parents reported that children engaged in about three different spatial activities and 11 different number activities across the 2 days. The mean time spent engaging in math activities over 2 days was 129.76 min, with a standard deviation of 106.02 min.

Table 6 shows the intercorrelations between the two count variables (number activities and spatial activities) and the duration of time spent engaging in math activities. Not surprisingly, children who engaged in more total math activities tended to do more of both types of activities. Looking at the correlation between the different types of activities, there was a moderately strong correlation between the diversity of children’s number activities and spatial activities. Additionally, the duration of time children spent engaging in math activities was moderately correlated with the diversity of math activities in which children engaged, including both number and spatial activities.

**Table 6:** Pair-wise correlations among measures of number, spatial, and overall math activities from time diary interviews.

	1	2	3
1. Diversity spatial activity			
2. Diversity number activity	0.66***		
3. Diversity of total math activities	0.74***	0.85***	
4. Minutes of math activities	0.50***	0.55***	0.46***

*p < 0.05, **p < 0.01,***p < 0.005.

### Intercorrelations across different methods of assessing math support and toddlers’ math skills

In our analysis we also examined interrelations across the multiple methods of assessing math support. We present intercorrelations between number and spatial activities separately (Tables 7, 8, respectively). In order to examine whether these measures are also related to toddlers’ early math skill, we correlated these measures with children’s counting and spatial reasoning skills (Tables 7, 8).

**Table 7:** Pair-wise correlations among number measures across all methodologies and toddlers’ number and spatial skills.

	1	2	3	4	5	6	7	8	9	10	11
1. Freq. number concepts (q)											
2. Freq. written numerals (q)	0.53***										
3. Freq. number applications (q)	0.38***	0.41***									
4. Talk number symbols (o)	0.11	0.13	0.02								
5. Talk counting (o)	−0.01	−0.04	−0.11	0.20*							
6. Talk labeling sets (o)	0.20*	0.11	0.12	0.38***	0.34***						
7. Total number utterances (o)	0.16	0.09	0.04	0.61***	0.68***	0.88***					
8. Diversity number activity(td)	0.31***	0.34***	0.23**	−0.03	0.12	0.16*	0.14				
9. Diversity of total math activities (td)	0.24***	0.25***	0.12	0.00	0.12	0.16	0.15	0.85***			
10. Minutes of math activities (td)	0.31***	0.23***	0.29***	−0.00	−0.03	0.08	0.04	0.55***	0.46***		
11. Counting	0.22*	0.23*	0.14	0.07	0.09	0.28**	0.23**	0.19	0.13	0.15	
12. Spatial skills	0.17	0.10	0.06	0.02	−0.02	0.06	0.03	0.21*	0.17	0.35**	0.29**

(q), survey home number activities, (o), number talk content from the semi-structured observations, (td), number activities reported by parents in the time diary interview. *p < 0.05, **p < 0.01,***p < 0.005.

**Table 8:** Pair-wise correlations among spatial measures across all methodologies and toddlers’ number and spatial skills.

	1	2	3	4	5	6	7	8	9	10
1. Freq. shape activities (q)										
2. Freq. building activities (q)	0.34***									
3. Talk shapes (o)	0.05	0.06								
4. Talk locations, directions, orientations (o)	0.07	0.18*	0.34***							
5. Talk deictics (o)	0.04	0.11	0.06	0.41***						
6. Total spatial utterances (o)	0.09	0.15	0.56***	0.83***	0.68***					
7. Diversity spatial activities (td)	0.24***	0.23**	0.17*	0.05	−0.06	0.09				
8. Diversity of total math activities (td)	0.18*	0.13	0.33***	0.06	−0.10	0.15	0.74***			
9. Minutes of math activities (td)	0.19*	0.16	0.14	0.07	−0.01	0.11	0.49***	0.46***		
10. Counting	0.05	0.04	0.02	0.15	−0.25**	0.01	0.02	0.13	0.15	
11. Spatial skills	−0.04	0.21*	0.14	0.18*	−0.15	0.12	0.11	0.17	0.35**	0.29**

(q), survey home number activities, (o), number talk content from the semi-structured observations, (td), number activities reported by parents in the time diary interview. *p < 0.05, **p < 0.01,***p < 0.005.

#### Number activities

The frequency of all three of the number activities asked about in the questionnaire (*number concepts, written numerals*, and *number applications*) were significantly and positively associated with the diversity of number activities endorsed in the time diary interviews, as well as with the duration of time spent doing math activities as reported in time diaries (Table 7). On the other hand, the observational measures of number talk had few correlations with the other number activity measures. The only type of number talk that was related to other number measures was *labeling sets*; it was positively correlated with the frequency activities involving *number concepts* and the diversity of number activities as reported *via* time diaries.

#### Spatial activities

We conducted similar analyses of interrelations among multiple data sources of parental support for spatial skills (Table 8). As with number activities, parents’ reports on spatial activities of the survey were correlated with time diary reports of spatial activities. In particular, the frequency of engagement in *shape activities* and *building activities* were positively and significantly related to the diversity of spatial activities reported in time diaries. Similar to number talk, spatial talk was largely unrelated to parents’ reports of spatial activities drawn from both the questionnaire and the time diary interview. The lone exceptions were a marginal relation between talk about *locations, directions and orientation* and the frequency of *building activities* and a marginal association between talk about *shapes* and the diversity of spatial activities reported in the time diary interviews.

#### Correlations with toddlers’ math skills

Lastly, we examined concurrent validity between the HME measures and children’s counting and spatial reasoning skills. For number activities (Table 7), the frequency of number concept activities and written number activities measured *via* questionnaire positively related to toddlers’ counting skills. From the observational tasks, total number utterances also were positively correlated with counting, and this seems to be driven primarily by talk involving *labeling sets*. Time diary measures were unrelated to counting skills. Number activities were largely unrelated to spatial skills, with the exception that the diversity of number activities and total minutes of all math activities reported in time diaries were positively associated with spatial reasoning skills.

Table 8 shows results of correlations between the spatial HME measures and counting and spatial relation skills. Spatial measures were mostly unrelated to early counting skills, with the exception of a negative correlation with utterances involving *deictics*. In contrast, spatial skills were related to spatial HME measures. From the survey, frequency of *building activities* was positively related to spatial skills. From the observational tasks, the number of utterances concerning *locations, directions, orientations* were positively correlated with spatial skills. Lastly, the duration of time spent engaging in math activities, as reported by parents in the time diaries, was also positively related to toddlers’ spatial reasoning skills.

## Discussion

This study examined the home math environment (HME) of 157 toddlers using three distinct methodologies: survey questionnaires, time diaries, and observations of math talk. Looking across all three methodologies, it is clear that the parents and toddlers in this sample were frequently engaging in math activities and math talk. Comparing the descriptive statistics observed here with those from a preschool sample with similar methods and measures (Bachman et al., 2020), we see very similar frequencies of HME among toddlers and preschool-aged children. For instance, both toddler parents and preschool parents in the Bachman et al. study reported a mean of 3.7 on the frequency of building activities in the survey. However, the families with toddlers generally displayed comparatively higher levels of HME engagement than the families with preschool-aged children in Bachman et al. (2020). Specifically, looking at survey items, preschool parents in the Bachman et al., study reported a mean of 2.5 on the 1–5 scale for frequency of all number activities aggregated, while the toddler parents here reported between 2.4 to 3.8 on the three number activity subscales included. Similarly, the diversity of number and spatial activities reported in the time diaries averaged about 6.5 and 1.5 activities, respectively, for preschoolers (Bachman et al., 2020). In this study, toddler parents reported nearly double the amount of activities across the 2 days: about 11 number activities and 3.4 spatial activities on average. This finding is not surprising since toddlers may spend more time in the home, as attendance in non-parental care grows dramatically from age 2 to ages 4–5 (from around 45 to 75%; U.S. Department of Education, National Center for Education Statistics, 2021). Moreover, given that the discrepancy is most apparent in time diary reports, this suggests that using time diaries to assess HME in toddlerhood may be especially useful.

The primary aim of the present study was to extend past work triangulating measures of the home math environment to two- and three-year-old children in order to understand the opportunities for developing number and spatial skills that toddlers experience at home. We find that measures that address the frequency and diversity of parent–child math activities, including traditional survey measures as well as novel time diary interview measures respectively, are moderately intercorrelated with one another, whereas measures of math talk drawn from direct observations of parent–child interactions seem to reflect a separate, independent component of the HME. Given at least some past work with toddlers and older children demonstrating that both math activities and math talk predict children’s math skills (e.g., Levine et al., 2010; Pruden et al., 2011; Daucourt et al., 2021), we argue that these dimensions are worthy of further exploration among younger children and stress the need for multimethod studies differentiate children’s opportunities to learn math. Indeed, our correlational analyses show that both components of HME, math activities and math talk, demonstrate unique patterns of association with different aspects of early math skills.

It is important to note that despite modest correlations between the survey measures of frequency of math activities and time diary measures reflecting diversity and duration of math activities, our results suggest that both methodologies have unique concurrent validity and may be important to incorporate in any comprehensive measure of the HME. This is particularly clear when looking at correlations between HME measures and children’s math skills. For instance, although the frequency of number activities (drawn from survey items) did not relate to spatial skills, the diversity of number activities and duration of math activities (drawn from time diaries) showed positive associations with spatial skills. It could be that the more comprehensive time diary prompts, which include example activities and are asked by trained interviewers, aid parents in recalling math-related activities that parents do not immediately think of as math activities when going through the survey items. In addition, the duration of math activities, which is only able to be accurately assessed *via* time diaries, was related to math skills.

### Differentiating math activities and math talk

Despite the fact that math activities and math talk both expose children to math content, we find little evidence that these aspects of the HME are associated. Specifically, parents’ use of number talk was not related to their reports of frequencies of number activities on either the survey or time diary measure. Likewise, parents who used more spatial talk with their children during a puzzle activity were not significantly more likely to engage in spatial activities at home. As such, we argue that engaging in frequent math activities and talking frequently about math reflect two unique methods of providing toddlers with opportunities to learn math in the early home environment. Math talk, which our results show relates to both early number and spatial skills, cannot be readily assessed *via* survey items. Indeed, math talk can occur during activities and interactions unrelated to math, like reading picture books, playing dolls, or playing outside. In our study, math talk occurred while children and parents engaged in pretend play involving the grocery store—an activity that is not inherently math-related and would not appear on a survey of home math activities.

Most research examining the HME in early childhood relies on measures of either parent–child math activities or parents’ math talk, and few studies have examined how these factors may or may not overlap. Among parents of older children, number talk was observed more frequently in number-related activities such as board games than in other activities such as play with dolls or action figures, but number talk still occurred in these non-numeric activities, especially for parents with higher levels of education (Thippana et al., 2020). Similarly, past work suggests that parents use more spatial talk during explicitly spatial activities (Ferrara et al., 2011; Verdine et al., 2019; Zippert and Rittle-Johnson, 2020). Based on these past findings, we would expect that parents who engage in more number activities would in turn use more number talk. However, our measures of number and spatial talk reflect how parents discuss this mathematical content when given the necessary time and materials to engage in these activities, which may not be true in everyday interactions in the home. Alternatively, our measure of math talk, which was based on the frequency of utterances that included number or spatial content, may not capture the most important aspects of these interactions. Other metrics of math talk, such as the complexity of these utterances, may yield more informative measures of children’s exposure to math content (Fox, n.d.).

In addition to extending this work to explore how parent reports of math activities and direct observations of parents’ math talk relate to toddlers’ math skills, there is also an open question regarding why parents might engage in one method of supporting math or another. There may be similar underlying characteristics that encourage or discourage a parent to engage in math activities and to talk about math, such that parents who report higher levels of math anxiety may select math activities less frequently with their children (e.g., Elliott et al., 2020) and also may discuss math concepts less often when interacting with their children (Berkowitz et al., 2021). However, given the lack of associations observed here, it is possible that factors that predict increased math talk may differ from those that predict engaging in math activities at home, particularly if parent–child activities reflect a more dyadic process and are shaped by structural constraints on families (e.g., Lleras, 2008; Bornstein, 2009; Snell et al., 2015; Elliott, 2020; Thippana et al., 2020). As such, engaging in math activities and math talk may represent two distinct approaches to supporting children’s math skills for families, and considering these different approaches may help inform interventions aimed at boosting the home math environment.

Correlations between math activities and math talk measures further underscore the importance of measuring both aspects of the home learning environment. Both frequency of number activities and number talk positively predicted toddlers’ counting and spatial abilities. And while neither diversity nor duration of math activities reported in the time diaries was associated with counting, both of these time diary measures were positively associated with early spatial skills. Looking across the associations between early math skills and all of the HME measures assessed here, our results suggest that all measures and methods of data collection provide valuable information regarding the home math environment and math development in toddlerhood.

### Alignment between number and spatial content

Across all three methods of data collection, aspects of the HME focused on number and spatial content could be differentiated, and yet we found that parents’ reports of number and spatial activities were moderately correlated, as were observations of number and spatial talk. For parent-reported survey measures, all intercorrelations among the three number factors and the two spatial factors reached statistical significance, and several of the strongest correlations were across number and spatial factors (e.g., shape activities and written numerals). Similarly, the correlation between the counts of different number and spatial activities from time diaries were also highly correlated. These findings are in line with previous reports of significant intercorrelations between number and spatial activities (Cahoon et al., 2017; Zippert and Rittle-Johnson, 2020), although others have reported no such associations (Hart et al., 2016; Purpura et al., 2020).

Additionally, parents’ uses of number and spatial talk were moderately correlated. Notably, the observations of number and spatial talk were drawn from distinct tasks, and so this association demonstrates that parents who use more spatial talk in a spatial task are also more likely to use more number talk in an unrelated task. In other words, this association may reflect a more general underlying tendency of parents to use number and spatial talk with their young children rather than a task-specific effect. Alternatively, it could be that some parents are just more talkative in general when interacting with their child. Stated differently, the parents that are using more number and spatial talk during the tasks may also be talking about non-math related content as well during the task. Future studies that examine multivariate predictors of parental math talk could control for total talk to inform this issue.

Importantly, our cross-domain associations between number and spatial talk seem inconsistent with previous findings by Lombardi et al. (2017). They found that mothers’ use of labeling set sizes during two different activities (playing with blocks and playing with a cash register and dress-up clothes) was unrelated to their support of learning spatial concepts while playing with blocks. However, the effect size in their study (*r* = 0.2) was very similar to the effect size in the present study (*r* = 0.19) suggesting that the larger sample size in our study (*n* = 157 compared to *n* = 140 in Lombardi et al.) may explain these discrepancies.

On the other hand, when looking at associations between number and spatial HME and children’s counting and spatial reasoning skills, cross-domain associations were infrequent (i.e., spatial HME predicting counting and number HME predicting spatial skills). Counting, which is an indicator of children’s early numeracy skills, was positively related to the frequency of number concept and written numeral activities as reported in the questionnaire and number talk (both total number talk and labeling in particular). Only one spatial HME measure was correlated with counting, and this was a negative correlation between math talk involving deictics and counting abilities. Although unexpected, deictics tend to be the simplest spatial location terms (e.g., “here,” “there”), and children with more advanced math skills likely understand more complex spatial location terms, like “below,” “underneath,” and “behind.” Accordingly, their parents may use fewer deictic words than the parents of children with worse math abilities, which would explain the negative relation between deictics and counting. Similarly, toddlers’ spatial skills were positively predicted by frequency of building activities and parent talk related to location, direction, or orientation, as well as duration of math activities reported in time diaries. As with counting, only one number HME measure related to spatial reasoning (diversity of number activities from time diary reports).

### Limitations

There are some limitations to this study that we must acknowledge. First, only one parent was observed with the toddler and responded to questionnaire and time diaries. This may underestimate the diversity and duration of math activities or math talk in the home environment if non-participating parents (or other people in toddlers’ lives) engage in math with the children. Second, the correlations between math activities and toddlers’ math skills may be obscured by the inclusion of only one parent’s math talk and report of math activities. Children that are experiencing rich home math environments, but mostly with the non-responding parent, may have strong math skills related to math activities that were not captured by our observational tasks or parent reports since they occur with the non-responding parent or other adult.

Also, participants in this study tended to be more sociodemographically advantaged than the U.S. population as a whole, with more than three-quarters of the parents in the sample being highly educated (having a bachelor’s degree or higher), married, and non-Hispanic White. Thus, results of this study may not generalize to a wider or more diverse population. This is especially true given documented associations in the literature between home learning environment and family socioeconomic status (e.g., Dearing et al., 2012; DeFlorio and Beliakoff, 2015; Galindo and Sonnenschein, 2015; Dearing et al., 2022). Accordingly, future studies must replicate analyses capturing and correlating surveys, time diaries, and observational measures of the HME with a larger and more diverse sample.

Lastly, this study uses cross-sectional data; all measures were drawn from a single window of children’s toddlerhood. Thus, we are unable to provide any information regarding whether observed associations between HME measures are stable or change over children’s development. Additionally, although we observed links between several of the HME measures and toddlers’ math skills, the cross-sectional nature of these data prevents us from making inferences regarding whether children’s HME experiences improve math skills, or, vice versa, whether toddlers with better math skills are inclined to engage in more math activities. Alternatively, the observed associations may be attributable to another, unobserved characteristic of children or families (Elliott et al., 2017; Thippana et al., 2020; Daucourt et al., 2021). Future research should explore these questions.

## Conclusion

In comparing three measures of the HME, we find that parental reports of frequency of children’s number and spatial activities on traditional survey measures correlate with a novel time diary approach to measure the diversity and duration of math activities. These findings are consistent with our past work with parents of preschoolers (Bachman et al., 2020) and highlight the potential utility of time diary measures for assessing the home math environment with less bias due to parental recall demands. More work is needed to explore this approach, however, and to compare predictive validity of time diary and survey measures of HME for children’s later math skills. Additionally, we find that parents’ talk about math during structured observations with their toddlers reflects a distinct, unrelated aspect of the home math environment, suggesting the need for more work exploring whether and how math talk in these interactions relates to children’s math learning. Future work is needed to assess differential, longitudinal prediction of children’s math skills over time across these various metrics, as well as to explore the characteristics of parents and children that explain individual differences in these behaviors. Nonetheless, these findings demonstrate the need for multimethod approaches to measuring the HME in toddlerhood in order to obtain a better understanding of the multitude of opportunities for learning math that young children experience in their daily lives.

## Data availability statement

The original contributions presented in the study are included in the article/supplementary materials, further inquiries can be directed to the corresponding author.

## Ethics statement

The studies involving human participants were reviewed and approved by Human Resource Protection Office, IRB, University of Pittsburgh. Written informed consent to participate in this study was provided by the participants’ legal guardian/next of kin.

## Author contributions

PM drafted sections of the introduction and discussion of the manuscript, oversaw the writing process, and edited the full document. LE drafted sections of the introduction and discussion of the manuscript, and assisted with data analysis. TP performed that data analysis, and wrote parts of the methods and results section. CP was a data collector, helped support the literature review for the manuscript, and compiled the bibliographic information. SD supervised the number talk coding team and wrote sections of the manuscript describing this measure. DF supervised the spatial talk coding team and wrote sections of the manuscript describing this measure. EV-D guided data analysis and interpretation of the study finding. EV-D, HB, and ML helped to conceptualize the manuscript and provided feedback and editing of the manuscript at different stages of development. LC coded all measures drawn from the time diaries. All authors contributed to the article and approved the submitted version.

## Funding

Primary funding for this research was provided by the National Science Foundation (Award number: 1920545) to HB, ML, and EV-D. This project also benefited from intellectual synergies stemming from several related studies funded by the Eunice Kennedy Shriver National Institute of Child Health and Human Development (1 R01 HD093689-01A1), an internal award from the Learning Research and Development Center at the University of Pittsburgh, and a Scholar Award from the James S. McDonnell Foundation to ML.

## Conflict of interest

The authors declare that the research was conducted in the absence of any commercial or financial relationships that could be construed as a potential conflict of interest.

## Publisher’s note

All claims expressed in this article are solely those of the authors and do not necessarily represent those of their affiliated organizations, or those of the publisher, the editors and the reviewers. Any product that may be evaluated in this article, or claim that may be made by its manufacturer, is not guaranteed or endorsed by the publisher.
